# Oxidative Injury in Ischemic Stroke: A Focus on NADPH Oxidase 4

**DOI:** 10.1155/2022/1148874

**Published:** 2022-02-03

**Authors:** Ganglei Li, Changsheng Ye, Yu Zhu, Tiesong Zhang, Jun Gu, Jianwei Pan, Feng Wang, Fan Wu, Kaiyuan Huang, Kangli Xu, Xiaomin Wu, Jian Shen

**Affiliations:** ^1^Department of Neurosurgery, The First Affiliated Hospital, College of Medicine, Zhejiang University, Hangzhou 310003, China; ^2^Department of Emergency, Anji County People's Hospital, Huzhou 313300, China; ^3^Department of Anesthesiology, Zhejiang Provincial People's Hospital, People's Hospital of Hangzhou Medical College, Hangzhou 310014, China

## Abstract

Ischemic stroke is a leading cause of disability and mortality worldwide. Thus, it is urgent to explore its pathophysiological mechanisms and find new therapeutic strategies for its successful treatment. The relationship between oxidative stress and ischemic stroke is increasingly appreciated and attracting considerable attention. ROS serves as a source of oxidative stress. It is a byproduct of mitochondrial metabolism but primarily a functional product of NADPH oxidases (NOX) family members. Nicotinamide adenine dinucleotide phosphate oxidase 4 (NOX4) is most closely related to the formation of ROS during ischemic stroke. Its expression is significantly upregulated after cerebral ischemia, making it a promising target for treating ischemic stroke. Several drugs targeting NOX4, such as SCM-198, Iso, G-Rb1, betulinic acid, and electroacupuncture, have shown efficacy as treatments of ischemic stroke. MTfp-NOX4 POC provides a novel insight for the treatment of stroke. Combinations of these therapies also provide new approaches for the therapy of ischemic stroke. In this review, we summarize the subcellular location, expression, and pathophysiological mechanisms of NOX4 in the occurrence and development of ischemic stroke. We also discuss the therapeutic strategies and related regulatory mechanisms for treating ischemic stroke. We further comment on the shortcomings of current NOX4-targeted therapy studies and the direction for improvement.

## 1. Introduction

Stroke is a cerebrovascular disease caused by organic brain injury, characterized by abrupt onset and rapid emergence of localized and disseminated deficits in brain function [1–4]. It is a disease with high disability, morbidity, and fatality rates [5–7]. Stroke can be classified as ischemic stroke or hemorrhagic stroke, with ischemic stroke accounting for approximately 80% of all cases [8, 9]. Ischemic stroke is a devastating cerebral disease that can cause permanent neurological damage [10]. Although the diagnosis and treatment of ischemic stroke have greatly improved [11–13], the prognosis of patients with ischemic stroke is still poor [14, 15]. Hence, it is necessary to further explore the pathophysiological mechanisms and therapeutic strategies for the treatment of ischemic stroke.

Oxidative stress plays a crucial role in the development and progression of many diseases, especially in ischemic stroke [16–22]. It results from an imbalance between the generation of ROS and antioxidant defense systems. Increased ROS could modify cell structure and enzymatic activity [23–25]. These changes lead to extravasation of blood components, increased inflammatory response, and even irreversible brain tissue damage [26]. Nicotinamide adenine dinucleotide phosphate (NADPH) oxidases (NOXs) and mitochondrial metabolism are the major sources of ROS [27, 28]. While ROS are byproducts of mitochondrial metabolism, they are functional products of the NOX family. NOXs are composed of the NOX1–5 and DUOX1–2 enzymes and play indispensable roles in cellular homeostasis [29], including gene expression, cell migration, proliferation, aging, and inflammation. NOX4, a member of the NOX family, was first identified in the kidney [30]. Growing evidence shows that NOX4 plays a salient role in many diseases. For example, NOX4 was found to function as an oncogene in non-small-cell lung cancer by enhancing glycolysis [31]. In addition, NOX4 facilitates cisplatin-induced acute kidney injury by regulating ROS-mediated programmed cell death and inflammation [32]. Notably, NOX4 is highly expressed in cerebral blood vessels and could contribute to ROS formation after acute cerebral ischemia. Emerging data suggest that NOX4 is significantly associated with ischemic stroke, particularly by increasing levels of oxidative stress [33]. Oxidative stress could contribute to ischemia- and hypoxia-induced brain injury and induce the formation of atherosclerosis, an important cause of ischemic stroke [34, 35]. Importantly, increasing studies demonstrate that the silenced NOX4 impairs BBB leakage, neuronal apoptosis, and oxidative stress. Researches on NOX4-targeted stroke treatment have made significant progress. NOX4 is a promising therapeutic target for treating ischemic stroke.

In this review, we explored basic information about MAGI2-AS3 and its subcellular location and summarized the literature describing the relationship between NOX4 and ischemic stroke, specifically its pathophysiological mechanism. In addition, we discuss therapeutic strategies targeting NOX4 for treating ischemic stroke.

## 2. Overview of NOX4: Physiological Function, Subcellular Location, and Expression Patterns

**Figure 1:**
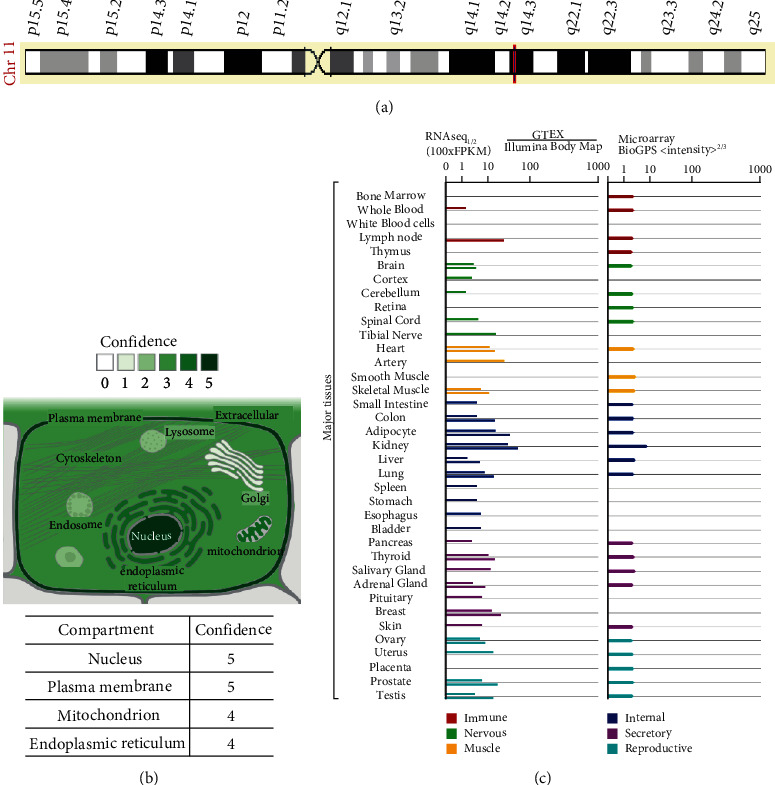
The subcellular localization and gene expression of NOX4 in human tissues. (a) NOX4 gene is located on chromosome 11q14. (b) The expression of NOX4 is concentrated in the nucleus, plasma membrane, mitochondrion, and endoplasmic reticulum. (c) The expression level of NOX4 in normal human tissues.

The *NOX4* gene is located on chromosome 11q14, as shown in Figure 1(a). Based on the GeneCard online database (https://www.genecards.org) [36, 37], NOX4 is widely distributed in cells, especially in the plasma membrane, nucleus, and mitochondria, as shown in Figure 1(b). The subcellular localization may be obviously correlated with the function of NOX4. In addition, the expression levels of NOX4 are normally elevated in the normal kidney, artery, lymph node, adipocyte, and breast tissues (Figure 1(c)). NOX4 has a close connection with the function and condition of multiple viscera and systems. More selective targeting of NOX4 is an important direction of stroke-associated researches.

The NOX protein family includes NOX1, NOX2, NOX3, NOX4, NOX5, and the dual oxidases DUOX1 and DUOX2 [29, 38]. NOX1–5 are expressed in all vascular cell types and play essential roles in many physiological processes, such as endothelial cell function, vascular permeability, and angiogenesis. NOX4 functions as a heme-containing catalytic subunit that contains six transmembrane domains [39, 40] and binds p22(phox) to form a heterodimeric enzyme complex [41–43]. Splice variants of the *NOX4* gene encode four proteins: NOX4B (62.7 kDa), NOX4C (25.7 kDa), NOX4D (31.8 kDa), and NOX4E (27.6 kDa), of which only the functionally active NOX4D is associated with the production of ROS [44–46]. While NOX4D lacks putative transmembrane domains, it has both NADPH- and flavin adenine dinucleotide-binding domains, which are required for electron transfer activity [45]. Upregulation of NOX4D expression promotes the production of NADPH-dependent ROS and thus enhances the phosphorylation of extracellular-signal-regulated kinase 1/2 and the nuclear transcription factor Elk-1 [45].

Some research groups have reported that NOX1, NOX2, NOX4, and NOX5 exist in all vascular cell types and directly affect many physiological processes, such as endothelial cell function, vascular permeability, and angiogenesis. Under pathological conditions, the expression of NOX4 is dominant in vascular endothelial cells, affecting both their proliferation and angiogenesis by increasing ROS production [47–49].

## 3. Pathophysiological Mechanisms of Ischemic Stroke

Stroke can be classified into ischemic stroke (IS) and hemorrhagic stroke that is characterized by abrupt onset and rapid emergence of localized and disseminated deficits in brain function [1, 2]. It is a cerebrovascular disease caused by organic brain injury. The most common type of stroke is ischemic stroke [3, 4]. Ischemic stroke was caused by supplying artery stenosis (the carotid and vertebral arteries) or occlusion or insufficient cerebral blood supply. Four types of ischemic stroke were recognized: TIA (transient ischemic attack), reversible ischemic neurological deficit (RIND), stroke in evolution, and completed stroke [50]. TIA has no infarcts, while RIND, stroke in evolution, and completed stroke have varying cerebral infarcts. Neurons lose their ability to maintain normal transmembrane ionic gradients after ischemia [50] and then cause a series of pathological changes, such as oxidative stress [51], excitotoxicity, inflammation [52], and apoptosis [53]. Increased ROS triggers oxidative stress after ischemia-reperfusion and then aggravates blood-brain barrier injury. These alterations cause blood constituent extravasation, escalate the inflammatory response, and lead to irreversible brain tissue injury [26].

### 3.1. ROS

Oxidative stress results from an imbalance between ROS generation and antioxidant defense systems [54, 55]. Various types of noxious stimuli lead to excessive production of ROS or reduce the antioxidative capacity and then cause tissue damage. ROS serves as a source of oxidative stress. It is a byproduct of oxygen metabolism but primarily functional products of NOX family members, including free radicals such as O_2_^−^ and OH and nonradicals such as H_2_O_2_ [56, 57]. The production of ROS is stimulated by a series of enzymes, such as cyclooxygenases, myeloperoxidases, and NADPH oxidase [58]. O_2_^−^, having less transmembrane capacity, achieves better transmembrane ability via the conversion of O_2_^−^ to H_2_O_2_. H_2_O_2_ produces ^·^OH through reacting with Fe^2+^ to undergo the Fenton reaction [59, 60].

**Figure 2:**
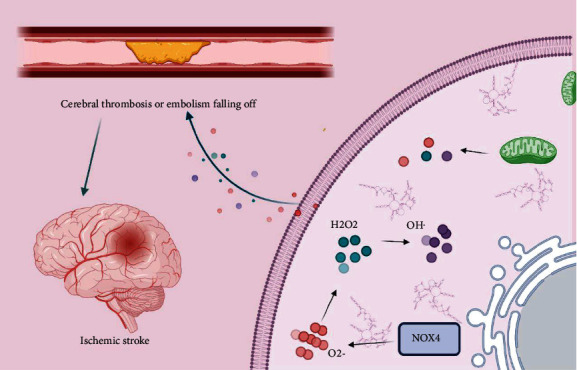
ROS serves as a source of oxidative stress. It is a byproduct of oxygen metabolism but primarily functional products of NOX family members, including free radicals such as O_2_^−^ and OH and nonradicals such as H_2_O_2_. Oxidative stress was closely associated with atherosclerosis, an important cause of ischemic stroke. Atherosclerotic plaque, very unstable, could lead to cerebrovascular blockage after the plaque is cracked or ruptured.

Increased ROS could degrade endocellular macromolecules (lipids, proteins, and nucleic acids) and lead to decreased enzymatic activity and alterations of cellular configuration [23–25]. Overproduction of ROS results in a decrease of cell membrane fluidity and permeability by induction of lipid peroxidation. Oxidative stress is not only involved in ischemia- and hypoxia-induced brain injury but was also closely associated with atherosclerosis, an important cause of ischemic stroke [34, 35]. ROS is known to play an important role in atherosclerosis progression [17, 61]. Risk factors for stroke could lead to vascular endothelial injury and facilitate penetration of LDL into the subendothelial space. LDL is oxidized by ROS [61] and becomes ox-LDL in the subendothelial space. Ox-LDL induces proinflammatory responses and vascular smooth muscle cell proliferation. Activated macrophages produce large levels of ROS by activating NADPH oxidase and then promote the formation of ox-HDL [62]. Finally, atherosclerotic plaque is formed and gradually hardens and narrows the arteries. Atherosclerotic plaque, which is very unstable, could lead to cerebrovascular blockage after the plaque is cracked or ruptured (Figure 2). Large amounts of ROS could induce the early inflammatory response and activate many kinds of immune cells [63]. Then, this triggers the release of numerous cytokines, generates a local effect, and further contributes to more generations of ROS. Oxidative stress and inflammation cross-promote each other, thus creating a vicious circle [64, 65].

The detection of oxidative stress and inflammatory reaction level potentially could have important clinical implications. Due to the short half-life and highly reactive nature [66, 67], it is difficult to detect the ROS level directly. 8-Hydroxydeoxyguanosine (8-OHdG) and malondialdehyde (MDA) are products of oxidative DNA damage and lipid oxidative damage and are not affected by metabolic and dietary factors. Hence, 8-OHdG and MDA are potential biomarkers of oxidative stress and could be used for the detection of the oxidative stress level [68–70]. It is an effective approach for the treatment of ischemic stroke by inhibiting ROS production or enhancing the ROS scavenging capacity. The expression levels of antioxidant enzymes were predominantly regulated by Nrf2 [71, 72]. Nrf2 has shown a neuroprotective effect in ischemic stroke [73]. It could be a potential biomarker for the treatment of ischemic stroke [74, 75]. Butylphthalide has been found to improve the prognosis of ischemic stroke via regulation of Nrf2 [76]. Due to the interaction between oxidative stress and inflammatory response, detection and interventions of inflammatory reaction could have positive significance for the treatment of ischemic stroke.

### 3.2. ROS and NOX4

While ROS are byproducts of mitochondrial metabolism, they are primarily functional products of NADPH oxidase (NOX) family members [77, 78]. Growing evidence indicates that NOX is one of the main sources of cellular ROS, including neutrophils and neurons [79–81]. Nox generates ROS by transferring electrons from NADPH to oxygen, resulting in severe oxidative stress [82, 83]. NOX4 is highly expressed in cerebral blood vessels [84]. Moreover, NOX4 is most closely related to the formation of ROS after acute cerebral ischemia. Dysregulation of NOX4 is involved in a variety of human diseases and was associated with a series of clinicopathological features. NOX4 has great potential as diagnostic biomarkers, prognostic indicators, and therapeutic targets. NOX4-generated ROS has played a significant role in the initiation and development of various human diseases, including ischemic stroke. NOX4 is involved in the onset of atherosclerosis by modulating macrophage cell death induced by oxLDL [85, 86]. On the other hand, oxLDL significantly enhanced NOX4 expression by activating the MEK1/ERK pathway, thereby increasing the production of ROS [87]. NOX4 recruited M2-type tumor-associated macrophages to promote tumor growth via regulating ROS/PI3K axis in non-small-cell lung cancer [88]. The role of NOX4 in ischemic stroke is attracting an increasing level of interest. Blockade of NOX4-mediated ROS production and signal transduction pathways may help to improve the prognosis of patients with ischemic stroke.

### 3.3. NOX4 and the Pathophysiology of Ischemic Stroke

Among NOX family members, NOX4, the most abundant vascular isoform, has shown potential as an important regulator of IR injury. It is significantly induced under hypoxic conditions in different tissues and cells, making it a promising therapeutic target for the treatment of ischemic stroke [89]. Accumulating evidence suggests that NOX4 suppression could reverse the effects of blood-brain barrier (BBB) breakdown, oxidative stress, and neuronal apoptosis during IR injury [33, 90]. In this section, we will further discuss the role of NOX4 in the pathogenesis of ischemic stroke.

#### 3.3.1. The Expression and Clinical Features of NOX4 in Ischemic Stroke

While expression levels of NOX4 are typically elevated in the normal kidney, artery, lymph node, adipocyte, breast, and brain tissue, high NOX4 levels in the brain contribute to ischemic stroke [90]. Some research groups have reported that the expression of NOX4 is high in endothelial cells, astrocytes, and neurons [91]. Further, the major source of NOX4 is endothelial cells in ischemic stroke [92]. *In vivo* experiments demonstrated that levels of NOX4 are significantly upregulated after cerebral IR injury [90, 93–96]. In addition, the *NOX4* rs11018628 polymorphism has been correlated with short-term recovery and decreased risk of ischemic stroke, but the molecular mechanism of this phenomenon remained to be unveiled [97].

#### 3.3.2. The Related Mechanisms of NOX4 in Ischemic Stroke

The expression of NOX4 plays a key regulatory role in the development of ischemic stroke. Several studies have revealed that NOX4 leads to ischemic stroke via BBB breakdown and neurodegeneration [33, 90]. Further studies showed that the knockdown of NOX4 impairs BBB leakage, neuronal apoptosis, and oxidative stress [98]. Lou et al. found that activation of the TGF-*β* signaling pathway facilitated the expression of NOX4 by upregulating the expression of ALK5 and SMAD 2/3 in ischemic stroke [99]. The suppression of ALK5 limited the expression of NOX4 and ROS production, resulting in the blockade of cerebral IR injury. The ACE2/Ang-(1-7)/Mas pathway is also able to ameliorate cerebral injury by downregulating the levels of NOX4 in ischemic stroke [100]. Bioinformatics predictions indicate that miR-29c-3p, miR-132-3p, and miR-29a-5p target the *NOX4* gene [101]. These microRNAs are thus promising biomarkers for the treatment of ischemic stroke.

## 4. Therapy for Ischemic Stroke

Stroke is a disease with high disability, high morbidity, and high fatality rates with no currently effective therapeutic strategies in the clinic. A growing number of studies have shown that NOX4 levels dramatically correlate with ischemic stroke. With an improved understanding of the pathogenesis and molecular mechanism of ischemic stroke, NOX4 appears to be an important regulator of ischemic stroke pathophysiology. NOX4 is also a promising therapeutic target for the treatment of ischemic stroke. In this section, we will discuss the treatment and related regulatory mechanisms of ischemic stroke (Table 1).

### 4.1. SCM-198

**Figure 3:**
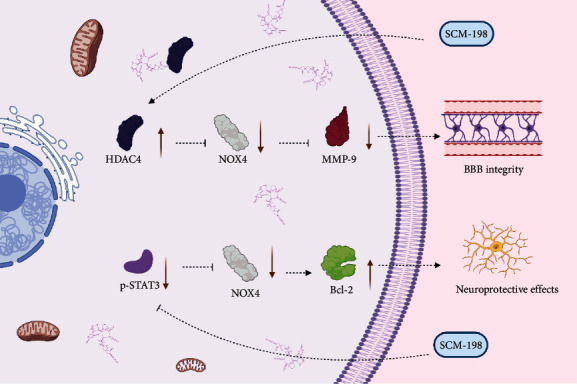
The function and molecular mechanism of SCM-198 in ischemic stroke. SCM-198 maintains the integrity of BBB integrity through the regulation of the HDAC4/NOX4/MMP-9 axis. SCM-198 significantly inhibits the expression of NOX4 to downregulate the level of MMP-9 by facilitating the expression of HDAC4. Moreover, SCM-198 plays a neuroprotective role via regulating the p-STAT3/NOX4/Bcl-2 pathway. SCM-198 promotes the expression of Bcl-2 through downregulating the expression level of p-STAT3 and NOX4.

Leonurine, also called SCM-198, is an alkaloid from the *Leonuri cardiacae herba* plant that is a promising effective treatment for several diseases [89, 111, 112], such as myocardial infarction, type 2 diabetes, and Alzheimer's disease [111, 113, 114]. Recent evidence indicates that SCM-198 also has potential efficacy against ischemic stroke [89, 102]. In a rat model of transient middle cerebral artery occlusion (MCAO), SCM-198 could reduce infarct volume and improve neurological deficits by maintaining BBB integrity after ischemic injury [102]. *In vitro* experiments showed that downregulating ROS production positively correlates with BBB integrity. Additional studies showed that SCM-198 facilitates BBB integrity by controlling the HDAC4/NOX4/MMP-9 axis (Figure 3). Moreover, SCM-198 was shown to play a neuroprotective role by regulating the p-STAT3/NOX4/Bcl-2 pathway, and the p-STAT3 protein was able to bind and regulate NOX4 directly [89]. SCM-198 might be an effective new agent for the treatment of cerebral ischemia and reperfusion injury. But the functional roles *in vivo* need more investigation into ischemic stroke.

### 4.2. Isoquercetin (Iso)

Iso, a novel plant extract [115], exhibits a wide range of therapeutic effects against cancer [116], acute myocardial infarction [117], and diabetes mellitus [118]. Recently, we found that Iso could decrease infarct volume and brain swelling and improve neurological deficits [73]. Iso inhibits oxidative stress by reducing ROS production and inducing the expression of the *SOD* and *CAT* genes. Both *in vivo* and *in vitro* experiments showed that Iso elicits antiapoptotic and antioxidant effects via Nrf2-mediated suppression of the NOX4/ROS/NF-*κ*B axis [73]. Silencing of Nrf2 was found to impair these effects of Iso. Therefore, Iso holds promise as a future treatment of ischemic stroke.

### 4.3. Ginsenoside Rb1 (G-Rb1)

G-Rb1, the major pharmacological extract from ginseng, has been reported to exhibit neuroprotective effects [103, 104]. In an animal model of MCAO, Chen et al. observed that G-Rb1 could reduce infarct volume, improve neurological deficits, and decrease the degree of cerebral edema. G-Rb1 was also shown to downregulate NOX4 expression and activity in ischemic stroke. Moreover, these authors found that G-Rb1 could maintain BBB integrity by inhibiting the neuroinflammation-stimulated induction of NOX4- and MMP-9-derived free radicals. However, these studies are limited by the small sample size. Large sample size *in vivo* research is still needed to further confirm the role of G-Rb1 in ischemic stroke. Additionally, the downstream regulatory mechanism of NOX4 needs to be understood further.

### 4.4. Electroacupuncture

Electroacupuncture, a type of Chinese medicinal therapy, is increasingly used to treat a variety of diseases, including as an adjuvant treatment for ischemic stroke [119–122]. Jung et al. [105] reported that electroacupuncture decreased infarct volume and improved neurological deficits after ischemic injury. These authors showed that electroacupuncture preconditioning markedly reduced the production of ROS and aquaporin 4 (a BBB component) in astrocytes. The expression of NOX4 was significantly inhibited, while levels of NOX2 were not obviously altered in electroacupuncture-preconditioned mice [105].

### 4.5. Betulinic Acid

Betulinic acid is a pentacyclic triterpene that is naturally found in birch trees [123–125]. It shows promise as a valuable treatment for cancer [126–128], nonalcoholic fatty liver disease [129], AIDS, and ischemic stroke [130, 131]. Betulinic acid has been shown to markedly attenuate infarct size and neuronal apoptosis and improve neurological deficits after IR injury [93]. Pretreatment with betulinic acid was shown to downregulate levels of NOX4 and ROS after cerebral ischemia. There are still some limitations in the study of betulinic acid and ischemic stroke. The effectiveness of the treatment was significantly noticed, but not as posttreatment after cerebral ischemia. It was also limited by a short period of observation. The long-term effects of betulinic acid remain unclear.

### 4.6. Nanomule Peptide Carrier

NOX4 is highly expressed in cerebral blood vessels and could contribute to ROS formation after acute cerebral ischemia. It could be a feasible way to reduce the expression of NOX4 by using NOX4-specific siRNA. It has been demonstrated that a twelve-amino acid peptide (MTfp), derived from melanotransferrin (MTf), could efficiently transport siRNA through the BBB model by forming a novel peptide-oligonucleotide conjugate (POC) [106, 107]. MTfp-NOX4 POC further reduced the expression level of NOX4. *In vivo* experiments showed that silenced NOX4 reduced infarct size and promoted neurological recovery after cerebral infarction. MTfp-NOX4 POC provides a novel insight for the treatment of stroke.

### 4.7. Other Therapeutic Strategies

Several studies have revealed that VAS2870, a NOX inhibitor, blocks neuronal apoptosis and oxidative stress by targeting NOX2 and NOX4 after ischemic injury [95, 98]. Intravenous injections of VAS2870 prior to reperfusion appear to significantly upregulate the levels of miR-29c-3p, miR-132-3p, and miR-29a-5p, which target NOX4 [101]. Li et al. [91] observed that melatonin may elicit antiapoptotic and antioxidant effects by suppressing the expression of NOX2 and NOX4 after cerebral ischemia. Safflor Yellow B, reducing neuronal injury after ischemia-reperfusion, was also involved in NOX4 regulation [108]. Guhong injection could significantly suppress NOX4 expression in the treatment of ischemic stroke [109]. In addition, the NOX inhibitor apocynin was demonstrated to improve neurological deficits in ischemic stroke [110]. Combinations of these therapies may also provide new approaches to treat ischemic stroke [132].

## 5. Conclusions and Future Perspectives

Ischemic stroke is a disease with high disability, high morbidity, and high fatality rates. Despite advancements in cancer treatment techniques, ischemic stroke remains a leading cause of death and disability worldwide. Therefore, it is urgent to find new diagnostic markers and treatments against ischemic stroke. This requires a deeper understanding of ischemic stroke pathophysiology. The crucial role of oxidative stress in the development of ischemic stroke is attracting widespread interest nowadays. Oxidative stress is not only involved in ischemia- and hypoxia-induced brain injury but was also closely associated with atherosclerosis. ROS is the core of oxidative stresses, but it is an unstable compound. So, the detection of stable oxidative stress products, 8-OHdG and MDA, is important for stroke diagnosis and treatment. The regulation of the ROS level may be an effective treatment approach for ischemic stroke. Oxidative stress was significantly associated with inflammatory reaction. Due to the close relationship of oxidative stress and inflammation, detection and interventions of inflammatory reaction may have a certain effect for the treatment of ischemic stroke.

ROS is the byproduct of mitochondrial metabolism but a main functional product of NOX family. In recent years, the role of NOX4 in stroke has been gradually brought to attention. High expression level of NOX4 is most closely related to formation of ROS after acute cerebral ischemia. NOX4 may be a potential diagnostic biomarker. This is an interesting topic, but it is still an emerging field. For clinical application, the biomarker must be stably expressed in body fluids (such as blood or urine or cerebrospinal fluid). Additionally, multicenter clinical research is required. In terms of treatment, clinical research about NOX4 has begun to make progress. The inhibition of NOX4 reversed the effect of blood-brain barrier breakdown, oxidative stress, and neuronal apoptosis during IR injury. Directly or indirectly targeted regulation of NOX4 could control the occurrence and development of ischemic stroke and improve the prognosis of patients. This field remains to be further studied. A promising drug must have the ability to stably regulate the NOX4 level and effectively transduce the effect. To reach this goal, we would need to conduct in-depth research on the structure and function of NOX4. Current research on targeting NOX4 is in its infancy, with many questions remaining. We need to conduct a more thorough analysis of the structure and function of NOX4 under physiological and pathophysiological conditions. In addition, the complex molecular mechanism that regulates the NOX4 network still needs to be further explored.

## Figures and Tables

**Table 1:** NOX4-related treatments and its associated mechanisms in ischemic stroke.

Drug name	Clinical features	Pathways	Refs
SCM-198	Infarct volume, neurological deficits	HDAC4, NOX4, MMP-9, p-STAT3, and Bcl-2	[89, 102]
Isoquercetin	Infarct volume, brain swelling, and neurological deficits	ROS, SOD, CAT, Nrf2, NOX4, ROS, and NF-*κ*B	[73]
Ginsenoside Rb1	Infarct volume, neurological deficits, and the degree of cerebral edema	NOX4 and MMP-9	[103, 104]
Electroacupuncture	Infarct volume and neurological deficits	ROS, aquaporin 4, and NOX4	[105]
Betulinic acid	Infarct size, neuronal apoptosis, and neurological deficits	NOX4 and ROS	[93]
MTfp-NOX4 POC	Infarct size and neurological deficits	NOX4	[106, 107]
VAS2870	Neuronal apoptosis and oxidative stress	NOX2, NOX4, miR-29c-3p, miR-132-3p, and miR-29a-5p	[95, 98, 101]
Safflor Yellow B	Infarct size, neurological deficits, and motor function	Ak046177, miR-134, NOX4, CREB, and Nrf2	[108]
Guhong injection	Infarct size and neurological deficits	PKC, HIF-1*α*, and NOX4	[109]
Melatonin	Neuronal apoptosis and oxidative stress	NOX2 and NOX4	[91]
Apocynin	Neurological deficits	NOX	[110]
